# Emotion and motion: superior memory for emotional but not for moving stimuli

**DOI:** 10.1007/s00426-025-02105-4

**Published:** 2025-03-24

**Authors:** Adam W. Cox, Paul Foret-Bruno, Inés Tchekemian Lanaspa, Isabella Zsoldos, Patrick S. R. Davidson, Hanna Chainay

**Affiliations:** 1 Laboratoire d’Étude des Mécanismes Cognitifs, Université Lumière Lyon 2, Lyon, France; 2 School of Psychology, University of Ottawa, Ottawa, Canada; 3 Laboratoire d’Etude des Mécanismes Cognitifs, Université Lumière Lyon 2, 5 Avenue Pierre Mendès France, Bron, 69676 France

**Keywords:** Emotional enhancement of memory, Dynamic superiority effect, Memory, Emotion, Movement

## Abstract

Two effects on memory have been described in the literature: the emotional enhancement of memory (EEM) (i.e., an emotional stimulus is better remembered than a neutral stimulus) and the dynamic superiority effect (DSE) (i.e., a moving visual stimulus is better remembered than a static stimulus). However, the DSE has previously only been studied using complex visual stimuli (e.g., video clips). Thus, the first objective of the present study was to examine whether the DSE will be observed with simple visual stimuli (i.e., isolated moving stimuli). The second objective was to examine whether people’s emotional memory will be affected by stimulus motion. We conducted three experiments, two using a free recall task, Experiment 1A (online) and 1B (in-person), and one using a recognition task (in-person). Participants viewed negative, positive, and neutral stimuli in two motion conditions, dynamic and static, and then had to recall or recognize them. In all three experiments, we observed an EEM but no DSE. Thus, our data verify that emotions affect memory performance but provide no evidence of motion effects on memory of simple stimuli.

## Introduction

Episodic memory (i.e., long-term conscious memory of past stimuli that includes information about what happened, where, and when (Tulving, 1972) can be influenced by many factors. One such factor is the emotional nature of the stimulus (e.g., Ack Baraly et al., 2017; Williams et al., 2022). Myriad previous research has found that emotional stimuli – whether they be words, sentences, virtual reality experiences, complex images, or simple objects – are more memorable than neutral ones (i.e., an “emotional enhancement of memory” [EEM] effect; e.g., Kensinger & Corkin, 2003; Mickley & Kensinger, 2008, Keightley et al., 2011; Chainay et al., 2012; Adelman & Estes, 2013; Gomes et al., 2013; Bowen et al., 2017; Salgado & Kingo, 2019; Cadet et al., 2022). Even if there still exists lively debate about whether this enhancement is identical for emotionally positive and negative stimuli (Williams et al., 2022), researchers have generally agreed that both types of emotionally valenced stimuli are more likely to be remembered than neutral ones.

Another factor that may enhance episodic memory is stimulus motion. Previous studies of motion and memory have used short film clips. Typically, participants have been shown film clips and static images extracted from the same sources, and later been asked to recognize them from among a mix of old and new stimuli. These studies have reported a “dynamic superiority effect” (DSE) in which a moving stimulus (i.e., a film clip) is easier to later recognize than its static counterpart (i.e., a still image from the same clip; Goldstein et al., 1982; Matthews et al., 2007; Buratto et al., 2009; Matthews et al., 2010; Candan et al., 2015). This DSE has been reported regardless of whether, in the recognition phase, the originally-moving stimulus was shown in motion (again) or not. Explanations of this effect have centered on the idea that stimulus motion is attentionally engaging and thus facilitates memory encoding (Matthews et al., 2010) and/or that moving objects contain more information than static ones (Buratto et al., 2009). However, video clips are very complex stimuli and the question remains whether this effect would be observed with more simple stimuli. Using more simple stimuli offers greater experimental control, and might help us isolate the cause(s) of the DSE. Simple objects can capture attention with simple motion (for example, Smith & Abrams, 2018; see also Levine & Edelstein, 2009). To our knowledge, however, no study has investigated this. Thus, the first objective of the present study was to answer the question: Does motion affect memory for simple stimuli?

Second, given the evidence that both emotion and motion may enhance memory, the question arises as to what effect they would have on memory if both were present at the same time. To our knowledge, the combined influence of these two factors on memory has not yet been investigated, at least not explicitly. The few previous studies interested in the effects of motion on memory have used only emotionally neutral material (Buratto et al., 2009; Candan et al., 2015; Goldstein et al., 1982; Matthews et al., 2007,2010). Conversely, although emotional film clips are probably easier to remember than neutral ones (for a review see Levine & Edelstein, 2009), to our knowledge only one study has included both static and moving emotional stimuli (Subramanian et al., 2014). In that study, participants initially viewed video clips (and static images) extracted from Hollywood-style movies. Then, their memory of a target object was tested using several multiple-choice questions. Interestingly, the authors found the opposite of the DSE as participants remembered significantly more details about the target object from the static images than from the video clips. A significant interaction between valence and motion condition was observed, showing that the general effect of motion condition was modulated by the valence of the stimuli: No significant effect of motion condition was observed for neutral stimuli, and only a marginally significant difference occurred for positive stimuli. However, it is not clear whether the effect of valence was observed for both static images and video clips because the paper does not report the relevant comparisons. Thus, the second objective of the present study was to further examine whether people’s emotional memory can be affected by stimulus motion and whether the effect is consistent across different emotional valences.

Given the counter-intuitive report from Subramanian et al. (2014) and the fact that there are no others of which we are aware, we therefore conducted three experiments to examine the effects of motion and emotion on memory for simple stimuli. Experiments 1A and 1B involved a free recall task, the former online and the latter in the laboratory. Experiment 2 involved a yes-no recognition task. Overall, we predicted that the emotional stimuli would be better remembered than the neutral ones (perhaps with the negative valence items being remembered particularly well; Bowen et al., 2018; Kensinger & Schacter, 2008) and that the moving stimuli would be better remembered than the static ones. Moreover, we thought it possible that the two effects (i.e., of valence and motion) may interact: the emotional-and-moving stimuli might be remembered particularly well.

## Experiment 1A

### Methods

#### Participants

The participants were University of Ottawa students recruited in February and March 2021, to fulfill a course requirement. All students willing to participate were included. The study was approved by the University of Ottawa Research Ethics Board and participants offered informed consent before beginning. One hundred and eighty-eight people initially took part in this study. Forty-three participants did not have their data recorded and had blank.*txt* files on the *JATOS* server (probably caused by incompatibility with certain internet browsers or attempts by a given participant to take part more than once). A further two participants were removed before data analysis because they had incomplete data sets that could not be interpreted for both static and moving conditions. Two participants were excluded before data analysis because their total recall score was below the 2nd percentile (> 2 SD), and one was removed because of possible duplication. The final number of participants included in the statistical analysis was 140, including 26 males and 114 females. Participants were aged between 17 and 37 years (M = 19.8, SD = 3.2).

We planned our sample size in advance, using G*Power (https://www.psychologie.hhu.de/arbeitsgruppen/allgemeine-psychologie-und-arbeitspsychologie/gpower). The recommended *n* was 44, based on a predicted medium within-subjects main effect size *f* = 0.25, with power = 0.95 and ɑ *p* =.05. We were mindful, however, that online testing might lead to more variability in our data, so to combat this we collected as many additional data as possible before the end of the semester.

### Materials and procedure

#### Stimuli

**Fig. 1:**
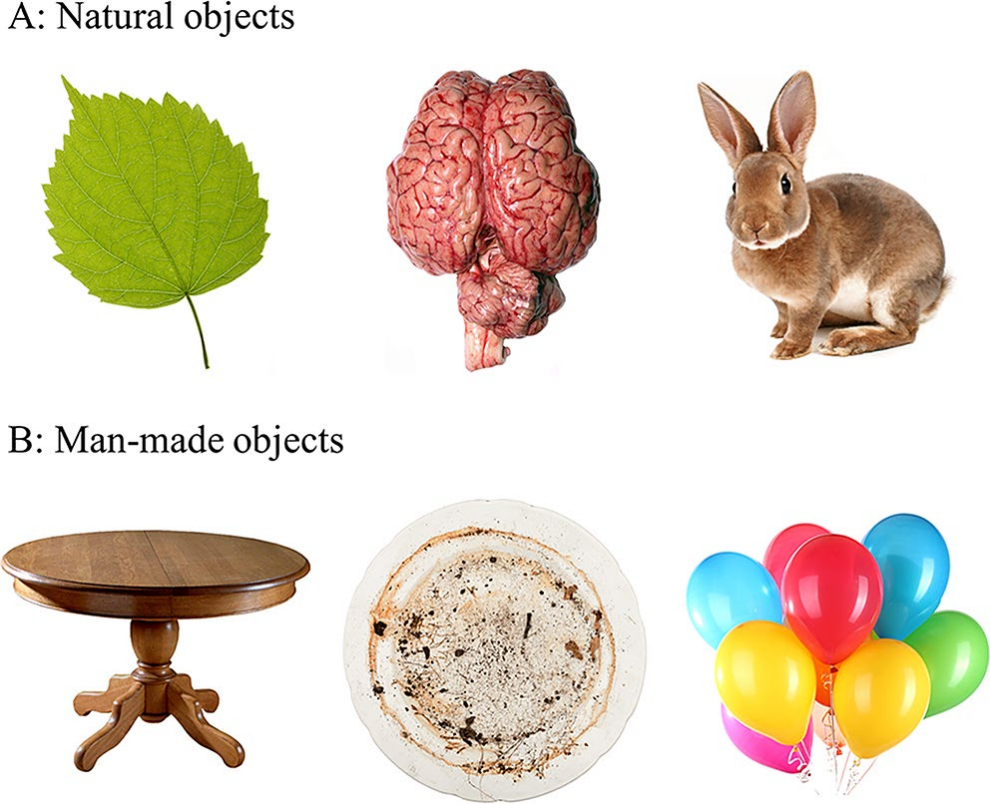
Example of stimuli, from left to right: neutral, negative, positive

The stimuli consisted of 60 color images of common objects (e.g., food, animals, furniture), some natural and others man-made, 20 images were negative, 20 were positive and 20 were neutral (see Fig. 1 for an example), and were presented to the participants either in moving or in static version. They were selected from the 988 images database previously pretested for valence and arousal in EMC laboratory^1^ by 30 young adults aged between 18 and 30 years (mean = 22.46, SD = 2.28). The valence and arousal were rated on 1 to 7 point scales, with 1 being very negative and 7 very positive on the valence scale, with 1 being not arousing and 7 highly arousing on the arousal scale. Stimuli were selected if their valence was between 1 and 3 as negative, between 3.5 and 4.5 as neutral and between 5 and 7 as positive. The arousal of the stimuli ranged from 2 to 4,6. The images were purchased from Shutterstock (https://www.shutterstock.com/fr/images*).* Stimuli from this set have previously been shown to elicit emotion effects on working memory (Chainay et al., 2023; Colliot et al., 2024).

The mean emotional valence of the negative stimuli (*M* = 2.61, *SD* = 0.318) was significantly different (*p* <.05) from the neutral (*M* = 4.11, *SD* = 0.300) and positive stimuli (*M* = 5.39, *SD* = 0.231), whereas the mean emotional valence of the positive stimuli was significantly different (*p* <.05) from neutral stimuli. The arousal of positive (*M* = 3.27, *SD* = 0.350), negative (*M* = 3.28, *SD* = 0.437) and neutral stimuli (*M* = 3.05, *SD* = 0.395) (all *p* <.05) was not significantly different. The items were split into two sets of 30 stimuli (10 negative, 10 neutral, 10 positive) and matched for emotional valence and semantic category (15 natural, 15 man-made) in purpose to show each set in static or moving condition to our participants. The composition of the sets never changed. These two sets were counterbalanced between static versus moving conditions for the different participants. Thus, one participant saw 60 images, half of them in static condition and the other half in moving condition. An additional eight neutral items were selected to absorb primacy and recency effects.

The moving stimuli were created by applying a transition animation to re-enact motion to the 60 coloured images described here above. Powerpoint (version 2204, Microsoft) animations “Fly in” and “Zoom” were used to make the item move linearly from one of the four corners on the screen and become larger as it progressed to the middle to re-enact the item approaching the participant, respectively. At the end of the movement, the size of the moving stimuli matched the static ones.

### Perceived stress scale (10-Item)

This study was carried out near the beginning of the COVID-19 pandemic when many peoples’ subjective stress levels were elevated. For this reason, we administered the Perceived Stress Scale-10 (PSS-10) (Cohen & Williamson, 1998). It contains ten items on a five-point Likert scale ranging from never (0) to very often (4).

### Procedure

**Fig. 2:**
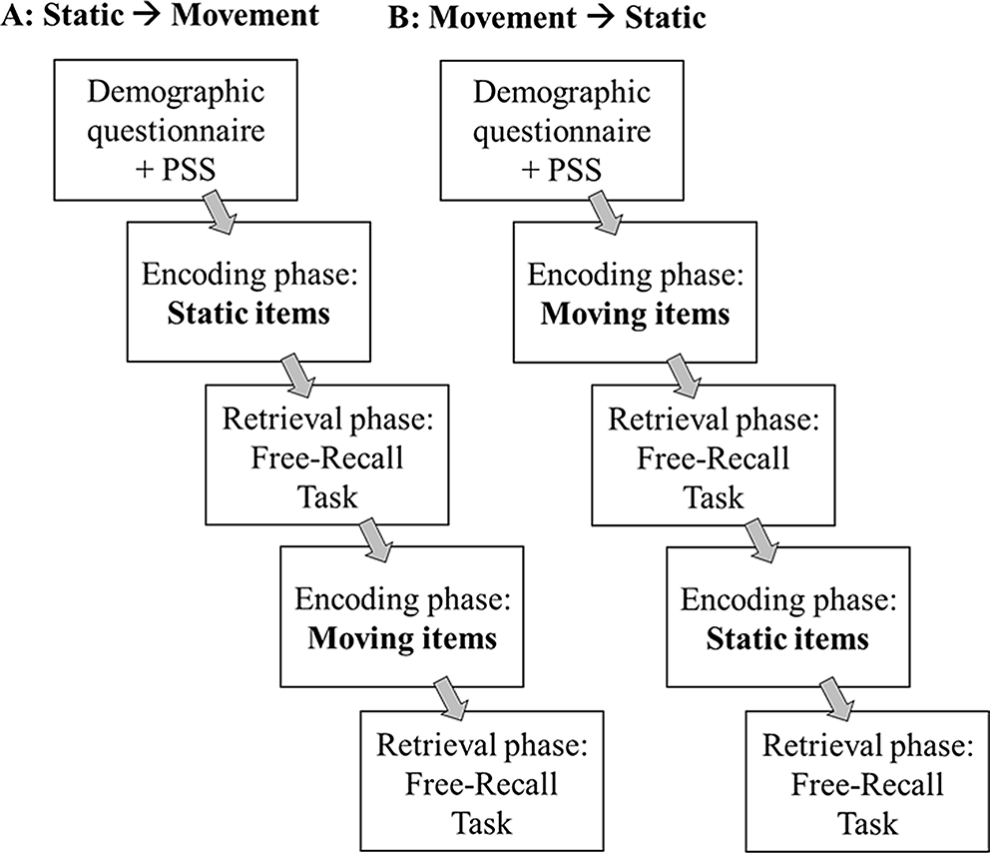
Experiment 1A Procedure

The study was completed in either English (*n* = 110) or French (*n* = 30) depending on each participant’s preference. Each participant completed two encoding phases followed, each, by a retrieval phase (free-recall task). The encoding was intentional, thus the participants were informed of a retrieval task. The encoding phases differed based on the condition of the stimuli (static or motion). We counterbalanced which set of images was used for the static versus motion conditions (e.g., if one participant saw set one in the static condition and set two in the moving condition, another participant saw set two in the static condition and set one in the moving condition). In addition, the order of the presentation of static and moving conditions was counterbalanced between participants (see Fig. 2). Thus, four counterbalanced versions of the experimental script were created. In each encoding condition (static, motion), each participant saw 30 images (10 positive, 10 negative, 10 neutral). To avoid primacy and recency effects, two control items were added at the beginning and the end of each encoding. These control items were not scored in the post-trial examination of participants’ free-recall data.

The experiment was provided to participants through a secure link hosted on the JATOS server. Participants read and signed a virtual informed consent form before beginning the experiment. Following the consent form, they completed a demographic form and the PSS-10. The demographic form consisted of questions regarding age, identified gender, and sex. The experiment began with the first encoding task either static or motion condition. To begin the encoding task the participant pressed the space bar key and passively watched as the stimuli appeared on the screen. Each stimulus was shown for 3 s followed by an interstimulus interval of 500 milliseconds. The sequence repeated until all 30 stimuli were shown before moving onto the first retrieval task.

During retrieval, participants were requested to list all items they remembered and any additional details they could recall (e.g., motion direction, color) in a multi-line entry text box. Participants then moved to the second encoding phase, followed again by a retrieval task. At the end of the second retrieval, participants were asked to disclose any breaks or distractions that may have occurred while completing the experiment.

### Data processing and statistical analysis

To quantify participants’ memory, a point was awarded for each accurately recalled item that the participant listed. An item was still scored as correct recall if the participant saw it in the first encoding phase but recorded it in the second free recall task. However, this carry-over of an item occurred very infrequently. Each recalled item was scored by two judges and the final score was a mean of the scores given by the two judges, one of whom was naïve to the details of the experiment. The data were then analyzed with JASP software. To investigate the effects of motion and valence on memory, we conducted a 2 × 3 repeated-measures ANOVA on the mean correct recall of items with the *Condition* (static, motion) and *Emotional valence* (negative, neutral, positive) as within-subjects factors. The normality of data distribution was checked with the Shapiro-Wilk test and sphericity with Mauchly’s test before running the ANOVA. The data of all experiments presented in this paper are openly available in Open Science Framework:https://osf.io/trbs8/?view_only=b1898f49ce724957840505aad690cb34.

### Results

#### Recall

ANOVA results showed a significant main effect of emotional Valence, *F(*2, 278) = 22.63, *p* <.001, η²*p* =.14. Planned comparisons were then performed to further investigate the differences between Valence categories, and these analyses revealed that the participants recalled significantly more negative items (*M* = 8.20, *SD* = 3.67) than neutral items (*M* = 6.68, *SD* = 3.57; *t*(278) = 6.67, *p* <.001) and positive items (*M* = 7.31, *SD* = 3.74; *t*(278) = 4.09, *p* <.001). Recall was also better for positive items compared to neutral items, *t*(278) = − 2.58, *p* =.01.

**Fig. 3:**
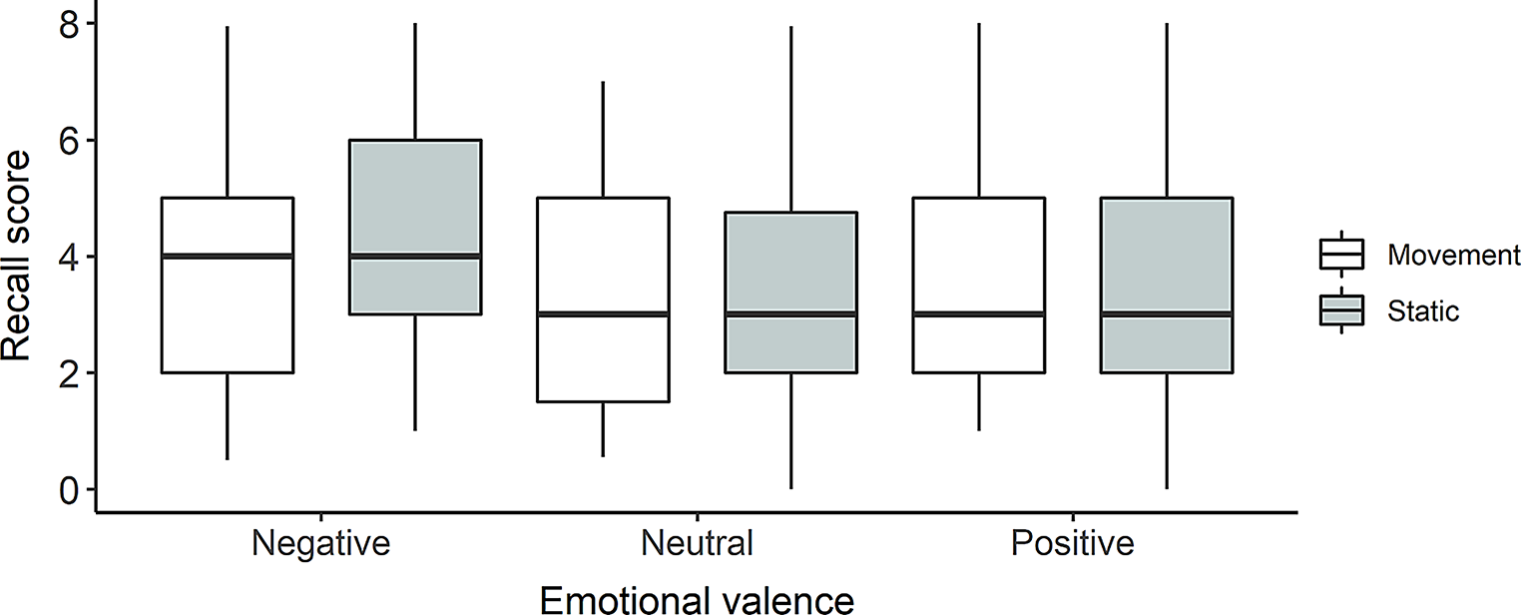
Recall score by Condition (motion, static) and Emotional valence (negative, neutral, positive) in Experiment 1A

The main effect of the condition was not significant, *F*(1, 139) = 1.75, *p* =.19, η²*p* =.01, as well as the interaction between Condition and emotional Valence, *F*(2, 278) = 2.58, *p* =.08, η²*p* =.02 (see Fig. 3).

#### Distraction

A large number of the participants (*n* = 66) answered that they were distracted while completing the study. The most common distractions were watching television, texting, listening to music, and conversing with someone else in the room. To test whether distraction could have influenced recall, we conducted a three-factor mixed two-way ANOVA, where the between-subjects factor was Distraction (distracted vs. non-distracted) and the within-subject factors were consistent with the previous ANOVA, Condition (static vs. motion) and Valence (negative, neutral, and positive). The results showed that the effect of distraction was not significant, *F*(1, 138) = 2.83, *p* =.10, and that distraction did not interact with Condition, *F*(1, 138) = 2.35, *p* =.13, or valence, *F*(2, 276) = 0.21, *p* =.81. Thus, possible distractions during the recall task did not notably influence recall.

#### Perceived stress scale

Overall, participants in this study were deemed to be moderately stressed at the time of participation (see Table 1). For comparison, Cohen and Janicki-Deverts (2012) found *n* = 223 individuals under the age of 25 had a mean score of 16.78 (SD = 6.86), and Denovan et al. (2019) found *n* = 524 university students had a mean score of 19.79 (SD = 6.37). The stress levels of our participants were significantly higher than both Cohen & Janicki-Deverts’ (2012) data, *t*(361) = 6.09, *p* <.001, *d* = 0.66, and Denovan et al.’s (2019), t(662) = 2.39, *p* =.02, d = 0.22. Correlational analysis collapsed across conditions (static/motion) found no significant correlation between PSS-10 score and the recall of negative, neutral or positive items (see Table 2).

**Table 1:** Mean scores of the questionnaires used in each experiment. Results are displayed as mean (SD)

Questionnaires		Experiment 1A	Experiment 1B	Experiment 2	
				Study phase	Test phase
PSS-10		21.26 (6.76)	20.75 (6.88)	23.10 (6.30)	23.30 (3.60)
BMIS	Pleasant/Unpleasant (out of 64)	-	47.28 (6.34)	46.86 (6.21)	44.66 (7.64)
	Aroused/Calm (out of 48)	-	27.70 (4.00)	28.31 (3.90)	27.52 (3.39)
HAD	Anxiety (out of 21)	-	-	9.39 (3.76)	9.41 (4.43)
	Depression (out of 21)	-	-	4.35 (3.59)	4.69 (3.56)

**Table 2:** Pearson correlations between stress, mood, anxiety and depression measures and recall/recognition for each Valence condition (negative, neutral, positive). *p <.05, **p <.01

			Experiment 1A	Experiment 1B	Experiment 2	
**Questionnaires**					Study phase	Test phase
PSS-10		Negative items	− 0.21	0.16	− 0.13	0.26
		Neutral items	− 0.21	0.14	− 0.07	0.24
		Positive items	− 0.10	0.05	0.16	0.28
BMIS	Pleasant/Unpleasant	Negative items	-	− 0.36*	0.12	0.30
		Neutral items	-	− 0.45**	0.16	0.31
		Positive items	-	− 0.60**	0.16	0.30
	Aroused/Calm	Negative items	-	0.09	− 0.09	− 0.12
		Neutral items	-	− 0.04	− 0.07	− 0.13
		Positive items	-	0.03	− 0.09	− 0.13
HAD	Anxiety	Negative items	-	-	− 0.012	− 0.095
		Neutral items	-	-	− 0.010	− 0.094
		Positive items	-	-	− 0.008	− 0.094
	Depression	Negative items	-	-	0.054	0.04
		Neutral items	-	-	0.056	0.04
		Positive items	-	-	0.056	0.04

## Discussion

The analyses revealed a significant effect of valence: emotional stimuli were better remembered than neutral stimuli. Within the emotional stimuli, we observed better recall for the negative stimuli than the positive ones, suggesting a negativity effect. No significant effect of Condition and no significant interaction effect between Valence and Condition were found. Finally, we also did not observe any significant correlation between participants’ stress level and recall, regardless of stimulus valence, suggesting that participants’ performance in the present study was not particularly linked to their mood or perceived stress.

In parallel to Experiment 1A in Canada, Experiment 1B was conducted independently in France. The original goal was to replicate the study in two different countries to verify that the effects of emotion and motion on memory would be the same. However, because of the different testing rules in France and in Canada during the COVID-19 pandemic, the administration of Experiment 1A was done online whereas Experiment 1B was done in person (including monitoring of performance). Thus, this difference also allowed us to examine whether these two different testing contexts would lead to the same results or not (e.g., Uittenhove et al., 2023).

## Experiment 1B

### Methods

#### Participants

Thirty-two French-speaking people were recruited to participate in this study, including 16 males and 16 females. Given our identical design and hypotheses, we carried forward our power estimates from Experiment 1A to here (note also that we expected variance to be smaller in Experiment 1B, because it was conducted in person). Participants were aged between 18 and 30 years (*M* = 25.78, *SD* = 2.68). They did not receive any money or academic credit for their participation. This study was conducted according to the Declaration of Helsinki Ethical Principles. Participants granted informed consent before taking part in the experiment and after having read the information notice.

### Materials and procedure

#### Stimuli

The stimuli for Experiment 1B were identical to Experiment 1A.

#### Brief mood introspection scale

In addition to completing the PSS-10 (see Experiment 1A for a brief description of the scale), participants also completed the Brief Mood Introspection Scale (BMIS; Mayer & Gaschke, 1988), which is often used in our laboratory. The BMIS is a 16-item scale used to compute four subscores: Pleasant-Unpleasant, Arousal-Calm, Positive-Tired and Negative-Relaxed mood, to assess the current mood of the participants via their own experience and meta-experience. Each item is an adjective referring to an emotional state (e.g., lively, gloomy). Participants evaluate to what extent this adjective matches their current emotional state using a Likert scale from 0 to 4. In the present study, only the scores for Pleasant-Unpleasant (score: min = 16, max = 64) and Arousal-Calm (score: min = 12, max = 48) subscales were computed using the reverse-scoring method, as we wanted to check for the possible influence of positive and negative mood and its intensity on memory performance (Ucros, 1989). The higher the score, the more pleasant and aroused the person is. This scale was used with the aim of observing whether participants’ overall mood would influence their recall.

#### Procedure

The experiment was run in a quiet laboratory room on a Dell laptop computer (diagonal monitor width of 17.3 inches). Participants first completed the two questionnaires. Then, they performed the encoding task (two blocks each after another) followed by the 5 min free-recall task. The free-recall was thus done only after viewing both conditions, static and motion. Here, participants had to verbally recall all the stimuli they remembered with as many details as possible. The experimenter recorded participants’ vocal responses with a cell phone.

The hypotheses of Experiment 1B were the same as those of Experiment 1A.

### Data processing and statistical analysis

The data processing and statistical analysis were conducted in the same way as in Experiment 1A.

#### Results

The results of the ANOVA showed a significant main effect of valence, *F*(2, 62) = 12.22, *p* <.001, η²*p* =.28. Planned comparisons were then performed to further investigate the differences between valence categories, and the results revealed that the participants recalled significantly more negative items (*M* = 8, *SD* = 1.92) than neutral items (*M* = 5.66, *SD* = 2.06; *t*(62) = − 4.60, *p* <.001) and positive items (*M* = 6.03, *SD* = 2.44; *t*(62) = − 3.87, *p* <.001). No significant differences were observed in recall between positive and neutral items, *t*(62) = − 0.74, *p* =.46.

**Fig. 4:**
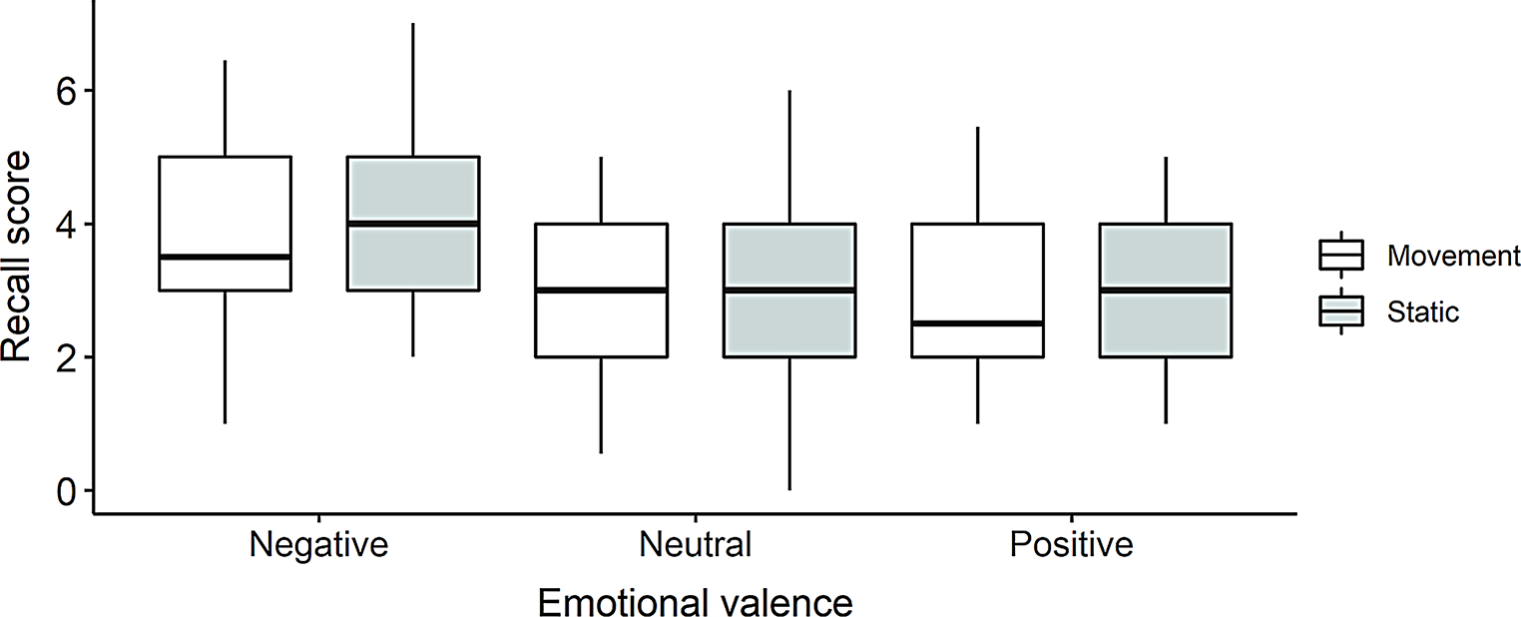
Recall score by Condition (motion, static) and Emotional valence (negative, neutral, positive) in Experiment 1B

The main effect of the condition was not significant, *F*(1, 31) = 1.42, *p* =.24, η²*p* =.04, as well as the interaction between condition and valence, *F*(2, 62) = 0.22, *p* =.80, η²*p* =.007 (see Fig. 4).

Total recall did not differ between online (Experiment 1A) and in-person (Experiment 1B) contexts (*p* >.05), suggesting that monitoring of performance did not influence participants’ recall.

#### Perceived stress scale

Participants in this Experiment were deemed to be moderately stressed at the time of participation (see Table 1). Correlational analysis collapsed across conditions (static/motion) found no significant correlation between PSS-10 score and the recall of negative, neutral or positive items (see Table 2).

#### Brief mood introspection scale

Mean scores for the Pleasant/Unpleasant and Aroused/Calm are reported in Table 1. A significant negative correlation was observed between the level of pleasantness and the recall of negative, neutral, and positive items (see Table 1). It would mean that the more the participants are in a pleasant mood, the less they remember items, regardless of valence: there is no evident explanation to this result. The level of arousal did not correlate significantly with the recall of negative, neutral, or positive items (see Table 2).

## Discussion

As in Experiment 1A, only a significant effect of Valence was observed in Experiment 1B. We also observed a better recall for negative items than positive ones, but interestingly, compared to Experiment 1A, positive stimuli were not better remembered than neutral stimuli. One possible hypothesis is that the smaller sample size of Experiment 1B prevented this effect from appearing. As in Experiment 1A, No significant effect of Condition and no significant interaction effect between Valence and Condition were observed either. Finally, there was no significant correlation between BMIS and PSS scores and recall, suggesting that participants’ performance in the present study was not particularly linked to their mood or perceived stress.

The surprising absence of an effect of motion on memory across our two experiments prompted us to design Experiment 2. Because the previous experiments that reported effects of motion on memory used a yes-no recognition task (Goldstein et al., 1982; Matthews et al., 2007, 2010; Buratto et al., 2009) the objective of our next experiment was to check whether the absence of an effect of motion in Experiment 1A and 1B could be due to the nature of the retrieval task that we used. Thus, in Experiment 2, the task was changed from free-recall to recognition. According to some authors, recognition and recall involve similar cognitive processes, but recognition of an item requires a “lower threshold of strength” for activation of a memory trace. Thus, recognition of an item is typically easier than recalling that same item (Kintsch, 1970). Thus, using a recognition task in the Experiment 2 would: (1) increase overall performance, which may demonstrate effects of motion as the previous studies that have showed these effects used recognition task (e.g., Matthews et al., 2010, Buratto et al., 2009), and (2) explore whether motion has a different influence on two different memory tasks, recognition and recall. Pilot testing of our task with a few different encoding/retrieval delays led us to use a one-week delay between encoding and retrieval in this Experiment in order to avoid a ceiling effect, mirroring what has been done in previous work (Matthews et al., 2007).

## Experiment 2

### Methods

#### Participants

Thirty-two French-speaking people were recruited among students of the Université Lumière Lyon 2 (Bron, France), including 26 females, 6 males and one “other”. Given our very similar design and hypotheses, we carried forward our power estimates from Experiments 1A and 1B to here (note also that we expected variance to be smaller in Study 1B, because it was conducted in person). Participants were aged between 18 and 23 years (*M* = 19.5, *SD* = 1.5). This study was conducted according to the Declaration of Helsinki Ethical Principles. Participants granted informed consent before taking part in the experiment and after having read the information notice.

### Materials and procedure

#### Stimuli

The material consisted of 120 stimuli extracted from the same set of 990 stimuli as Experiments 1A and 1B. The 60 stimuli were the same as Experiments 1A and 1B and 60 new stimuli were added in order to be able to run the recognition task. Stimuli were divided into three emotional valence categories (neutral: *M* = 4.05, *SD* = 0.35; positive: *M* = 5.39, *SD* = 0.22; and negative: *M* = 2.45, *SD* = 0.41), with 40 in each. For each stimulus, a static and a moving version were created. The creation of the moving versions followed the same procedure as described in Experiment 1A. Emotional valences were significantly different between the three categories (*p* <.001 for each comparison). Arousal was significantly higher for negative (*M* = 3.45, *SD* = 0.58) and positive (*M* = 3.30, *SD* = 0.34) stimuli compared to neutral ones (*M* = 2.75, *SD* = 0.57; *p* <.001 for each comparison), and was matched between positive and negative stimuli (*p* >.05). The stimuli were separated into two sets of 60 stimuli, Set 1 (valence: M = 4.07, SD = 0.34; arousal: M = 2.85, SD = 0.51) and Set 2 (valence: M = 4.03, SD = 0.37; arousal: M = 2.66, SD = 0.62). As in Experiment 1A and 1B each set comprised a subsets of 30 moving and 30 static stimuli that were each divided into 10 neutral, 10 positive and 10 negative stimuli. The two sets were matched for valence and arousal (*p* >.05 for each comparison). The Set 1 and 2 were used in counterbalanced manner in both encoding and retrieval task (e.g., Set 1), or only in retrieval tasks (e.g., Set 2) for one participant and in a reverse manner for another participant. For one participant, the stimuli were presented in the same version (static or moving) in encoding and retrieval phase.

#### Hospital anxiety and depression scale

In addition to completing the PSS-10 and BMIS (see Experiments 1A and 1B for a short presentation of the PSS-10 and BMIS, respectively), participants completed the Hospital Anxiety and Depression Scale (HAD; Zigmond & Snaith, 1983). This 14-item scale is meant to be used in hospital and clinical contexts to detect states of depression and anxiety in patients. It uses Likert’s scales from 0 to 4 to compute two scores corresponding to anxiety symptoms and depressive symptoms, assessed by 7 items each. With a highest possible score of 21, a score of 7 or less indicates an absence of symptomatology, a score comprised between 8 and 10 indicates a suspicious symptomatology, and a score of 11 or more indicates a definite symptomatology. This scale was used in order to have a more objective measure than the participants’s self-report of their possible mood disturbance.

#### Procedure

For Experiment 2, we used a recognition task. The stimuli were presented with Lab.js (https://lab.js.org/), Henninger; offline data collection) on a Dell laptop computer (diagonal width of 17.3 inches). Participants completed the three questionnaires before each experimental phase (study and test; see below).

The recognition task was divided into two phases, the study (encoding) and the test (retrieval), separated by a one-week delay. During the study phase, participants were shown 60 stimuli consisting of a block of 30 static stimuli and a block of 30 moving stimuli and were informed that they would be tested on their ability to remember them one week later. Each block comprised 10 stimuli from each emotional valence category. The order of apparition of the stimuli was completely randomized within each block. For each trial, a 500 ms fixation cross was displayed in the center of the screen followed by a 3-s presentation time of the stimulus. As in Experiments 1A and 1B, two items were placed at the beginning and the end of the study phase in order to control for primacy and recency effects. During the test phase, participants saw 120 stimuli, consisting of 60 old stimuli (i.e., shown in the study part) and 60 new stimuli. For each of the 120 stimuli, the participant had to click on a “yes” button if he or she thought that it was presented in the study phase, or on a “no” button if he or she thought it was not presented in the study phase. Participants had no limit of time to answer. The study part was performed exclusively on site, while the test part was done remotely for nine participants via a video conference software. During the video conference, the participants were emailed a link to run the task on their computer. The experimenter monitored the experiment remotely via the shared screen. Considering that overall recall performance did not differ between online and in-person contexts in the previous experiments, we chose to allow this flexibility to maximize the chances to respect a strict one-week delay between the two phases. Three counterbalancing orders were made to eliminate any presentation bias. First, during the study phase, half of the participants saw the static block first followed by the moving block, while the reverse was true for the other half. In the test phase, the presentation order of the static and moving blocks of the study phase was respected for each participant. Next, half of the participants saw Set 1 during the study phase, while the other half saw Set 2. Finally, as each stimulus had both a static and a moving version, half the participants saw the static version of Set 1 and the moving version of Set 2, while the other half saw the moving version of Set 1 and the static version of Set 2. All of this resulted in eight versions of the experimental script that were evenly distributed between the 32 participants.

The same hypotheses were made for Experiment 2 as for Experiments 1A and 1B.

### Data processing and statistical analysis

Data from three participants were excluded because of a misunderstanding of the task instructions. The number of Hits (i.e., saying “yes” for a stimulus that was presented in the study phase) and False Alarms (FA; i.e., saying “yes” for a stimulus that was not presented in the study phase) was counted for each participant, and the d’ index was calculated from these values. Data were then analyzed with JASP software. To investigate the effects of motion and valence on memory, we conducted a 2 × 3 repeated-measures ANOVA on mean d’ indices with the *Condition* (static, motion) and *Emotional valence* (negative, neutral, positive) as within-subjects factors.

## Results

The ANOVA revealed a significant main effect of valence, *F*(2, 56) = 3.32, *p* =.04, η²*p* =.11. Planned comparisons showed that recognition was significantly better for negative items (*M* = 1.35, *SD* = 0.86) compared to neutral items (*M* = 1.1, *SD* = 0.76; *t*(56) = − 2.10, *p* =.04) and positive items (*M* = 1.08, *SD* = 0.67; *t*(56) = − 2.35, *p* =.02). No significant difference was observed between positive and neutral items, *t*(56) = 0.24, *p* =.81.

**Fig. 5:**
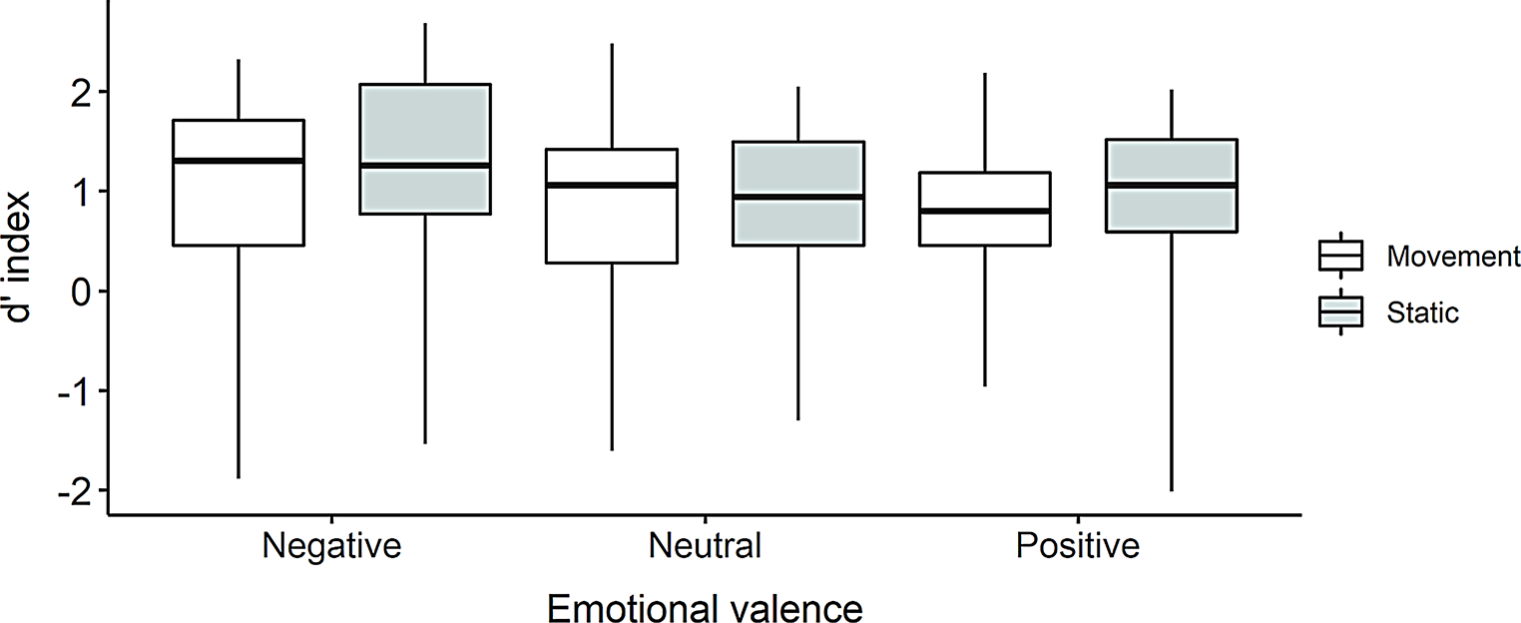
d’ index by Condition (motion, static) and Emotional valence (negative, neutral, positive) in Experiment 2

Finally, the main effect of condition was not significant, *F*(1, 28) = 1.06, *p* =.31, η²*p* =.04, as well as the interaction between condition and emotional valence, *F*(2, 56) = 0.28, *p* =.76, η²*p* =.01 (see Fig. 5).

### Perceived stress scale

On average, participants in this experiment were deemed to be moderately stressed both in the study phase and in the test phase (see Table 1). Correlational analysis collapsed across conditions (static/motion) found no significant correlation between PSS-10 score and the recognition of negative, neutral or positive items, for either the study or test phase (see Table 2).

### Brief mood introspection scale

Mean scores for the Pleasant/Unpleasant and Aroused/Calm for both study and test phase are reported in Table 1. The level of pleasantness did not correlate significantly with the recall of negative, neutral, or positive items, and that for the study phase and for the test phase. The level of arousal did not correlate significantly with the recall of negative, neutral, or positive items, and that for the study phase and for the test phase (see Table 2).

### Hospital anxiety and depression scale

Overall, participants in this study were deemed to have possible anxiety symptoms and no depressive symptoms both during the study and the test phase (see Table 1). The level of anxiety symptoms did not correlate significantly with the recall of negative, neutral, or positive items, and that for the study phase and for the test phase. Concerning the level of depressive symptoms, they did not correlate significantly with the recall of negative, neutral, or positive items, both for the study phase and for the test phase (see Table 2).

## Discussion

As in Experiments 1A and 1B, only a significant effect of Valence was observed in Experiment 2, resulting in better recognition of negative items compared to positive and neutral items. No significant effect of Condition or interaction between Valence and Condition were observed. The hypothesis that the absence of an effect of condition was due to the use of a free-recall task in Experiment 1A and 1B, contrary to all other studies on the same subject involving recognition tasks (Goldstein et al., 1982; Matthews et al., 2007, 2010; Buratto et al., 2009; Candan et al., 2015), is not supported. Finally, there was no significant correlation between questionnaire scores (BMIS, PSS, HAD A and HAD D) and the d’, suggesting that participants’ performance in the present study was not strongly linked to their mood, stress, or anxiety level.

### General discussion

In this study, we conducted three complementary experiments to investigate whether a ‘DSE’ is present in memory for pictures of simple objects (e.g., animals, tools, furniture, etc.), and whether it interacts with the ‘emotional enhancement of memory’ effect. The first two experiments involved a free-recall task (the first online and the second in the lab), and the third experiment involved a yes-no recognition task. All three experiments revealed a significant effect of emotion on memory, with better retrieval of negative items compared to neutral and positive items. Also, recall for positive items was significantly better than for neutral items in Experiment 1A only. However, we did not observe any significant effect of motion or interaction between valence and motion in any of the three experiments.

### Effect of emotion

Participants’ tendency to better remember emotional items than neutral items is congruent with many previous findings showing an EEM effect (e.g., Kensinger & Corkin, 2003; Mickley & Kensinger, 2008, Keightley et al., 2011; Chainay et al., 2012; Adelman & Estes, 2013; Gomes et al., 2013; Bowen et al., 2017; Salgado & King 2019; Cadet et al., 2022). However, it is interesting to note that this effect of emotions on memory tended towards a *negativity* effect in all three experiments, with negative items being significantly better remembered than both neutral and positive ones. Although there is still debate as to whether the effect of negative and positive emotions on memory is similar, this result appears in line with what has been suggested by Baumeister et al. (2001), that negative materials seem to undergo a more thorough processing at encoding, leaving a more complex memory trace compared to positive material (see Williams et al., 2022 for a review).

It is worth noting that the *negativity* effect was observed in both type of memory retrieval, recall (Experiment 1A and 1B) and recognition (Experiment 2), as previous work has suggested that in general the effects of emotion on memory might be weaker, or even absent, in recognition tasks compared to a free-recall task (e.g., see Kensinger & Schacter, 2008; and Kensinger & Fields, 2024, for reviews).

To sum up, we replicated the EEM effect in all three studies (albeit, for the negative stimuli), regardless of whether experiments were conducted online or in-person, and whether retrieval came immediately after encoding or one week after. As mentioned in the Introduction, the EEM is well-established using many different kinds of stimuli, and replication of it here serves as a validation of our methods in the present study. In contrast, the possible effects of motion are less clear, in particular using the simple stimuli that we employed here.

### Effect of motion

Surprisingly, we did not find an effect of motion on memory in any of the three experiments. This contrasts with what has been reported in the literature about a DSE (Goldstein et al., 1982; Matthews et al., 2007, 2010; Buratto et al., 2009; Candan et al., 2015).

Previous findings of motion effects have generally tested memory using a yes-no recognition task (Goldstein et al., 1982; Matthews et al., 2007, 2010; Buratto et al., 2009). This led us to hypothesize that the use of a free-recall task in the first two Experiments could have been an explanation accounting for the difference between our findings and previous ones, leading us to conduct Experiment 2. However, the use of a recognition task did not reveal any effect of motion either, which does not support this hypothesis.

Another explanation could come from the stimuli that were used. Previous studies that have found a motion effect have used more complex images or films (Goldstein et al., 1982; Matthews et al., 2007, 2010; Buratto et al., 2009; Candan et al., 2015), especially scenes involving people and perhaps more particularly faces (e.g., Candan et al., 2015, & Matthews et al., 2007), which involve further levels of encoding. This difference of complexity between these studies and ours could provide an explanation as to why no ‘DSE’ was obtained, as we used simple images of objects. The presence of complex and salient stimuli, such as bodies, faces or social interactions in film clips could be a facilitating condition for the apparition of an effect of motion on episodic memory, perhaps through a more thorough processing at encoding or through an increase in emotional valence caused by the motion. However, it has to be noted that one study that used film clips did not show the ‘DSE’ on memory performance (Subramanian et al., 2014). Thus this suggestion needs further empirical examination.

Furthermore, it has been suggested that each characteristic of an encoded stimulus, such as the details of its content, the relations between its different elements or the context in which it was encoded, makes its trace in memory more complex and specific (Tulving & Thomson, 1973; Versace et al., 2018). It is possible that motion could be one of these characteristics that confer to the stimulus a ‘mnesic advantage’ over a static stimulus, via a more complex memory trace, rendering it more salient and retrievable.

In our study, the use of repetitive, simple motions (i.e., diagonal lines) could have compromised the apparition of a ‘mnesic advantage’ for moving stimuli over static ones, making their accessibility in participants’ memory similar to static ones. This could explain the absence of any effect of motion on memory. Conversely, in studies that have used film clips (Goldstein et al., 1982; Matthews et al., 2007, 2010; Buratto et al., 2009), the complexity of the motion could have effectively provided stimuli with a ‘mnesic advantage’ over static images. For example, Candan et al. (2015) suggested that because film clips have a spatio-temporal context and motion has a meaning, it can lead to better encoding, whereas in our experiments the motion was not-specific and therefore meaningless. In addition, because the motions we used were very repetitive, participants’ attention was perhaps not focused enough on the stimuli and their motion which prevented better encoding of the moving stimuli (Matthews et al., 2010).

To sum up, we did not replicate the previously reported effect of motion on episodic memory using simple images of objects both in a free-recall task and a yes-no recognition task.

### Interaction between effects of emotions and motion

We also sought to explore in the present study whether the EEM effect and the ‘DSE’ interact. However, no significant interaction was found in all three experiments.

Two explanations could be put forward to account for the absence of an interaction. First, it is possible that the lack of statistical significance from the main effect of motion prevented any interaction effect from appearing. In that case, the question remains open as to whether the two effects interact, a question that would need to be explored in future studies. A second possibility could lay in the type of motion used in our study. Results from Poidevin et al. (2012) suggested that the type of motion of a visual stimuli (e.g., linear, undulatory, parabolic) can bear an emotional content in itself. In particular, in this study, participants attributed a neutral valence to simple linear motions, as opposed to undulatory and parabolic motions that were judged as positive and negative, respectively. Considering that in the present study we precisely used a simple linear motion, it is possible that this could have prevented an interaction effect between emotions and motion from appearing. Future work could explore this hypothesis by including the type of motion used for moving stimuli as an independent variable.

### Limitations

Several limitations of this study can be mentioned. First, sample sizes for Experiments 1B and 2 were relatively small and composed primarily of young people, most of whom were students. This limits the generalizability of the results. Further, although we examined anxiety and depressive symptoms in relation to memory in our participants, we did not specifically examine differences between those who had received a medical diagnosis of either and those who had not. Also, while the combination of the three experiments brings valuable insights concerning our research enquiry, differences in cultural background, language, and context may limit the comparability of the results. Future research could address these limitations by testing participants in a strictly identical context and by increasing sample size.

## Conclusion

This study sought to explore whether the ‘DSE’ is present in the recall and recognition of simple visual stimuli and whether it interacts with the well-known EEM effect using three complementary experiments. Our results suggest that, even though an effect of emotions on memory was obtained with simple visual stimuli, the ‘DSE’ is limited to more complex, meaningful stimuli. The question remains open as to whether this effect of motion interacts with the effect of emotions on episodic memory.

## Data Availability

The data that support the findings of this study are openly available in Open Science Framework at: https://osf.io/trbs8/?view_only=b1898f49ce724957840505aad690cb34.

## Footnotes

1 Laboratoire d’Etude des Mécanismes Cognitifs, Université Lyon 2, France.
